# Evaluating the efficacy of right vertical infra-axillary thoracotomy vs. standard median sternotomy for atrial septal defect repairs: A single center analysis from Pakistan

**DOI:** 10.12669/pjms.42.4.14416

**Published:** 2026-04

**Authors:** Mujeeb-Ur-Rehman, Hammad Saif, Muhammad Ihtesham, Muhammad Abdullah Ali

**Affiliations:** 1 Mujeeb-Ur-Rehman Congenital & Pediatric Cardiac Surgeon, Peshawar Institute of Cardiology, Peshawar, Pakistan; 2 Hammad Saif, Medical Student, Khyber Medical College, Peshawar, Pakistan; 3 Muhammad Ihtesham Post-Graduate Resident, Cardiac Surgery, Peshawar Institute of Cardiology, Peshawar, Pakistan; 4 Muhammad Abdullah Ali, Medical Student, Khyber Medical College, Peshawar, Pakistan

**Keywords:** Atrial Septal Defect, Cardiac Surgery, Median sternotomy, Thoracotomy

## Abstract

**Objective:**

To compare early postoperative outcomes of Right Vertical Infra-Axillary Thoracotomy (RVIAT) versus Standard Median Sternotomy (SMS) for atrial septal defect (ASD) and partial atrioventricular septal defect (AVSD) repair.

**Methodology:**

A retrospective study was conducted from June 2024 to July 2025 at the Peshawar Institute of Cardiology. Patients were divided into SMS (n=36) and RVIAT (n=36) groups. Operative times, hematologic parameters, transfusion requirements, ventilation duration, drainage volume, ICU/hospital stay, and complications were analyzed. Multivariate regression was used to adjust for age differences.

**Results:**

The SMS group was significantly older than the RVIAT group (18.39 ± 11.65 vs. 6.80 ± 5.91 years; p<0.001). SMS showed longer cross-clamp time and higher ventilation time, chest drainage, and blood transfusion requirements. After adjusting for age, RVIAT retained lower blood transfusion volume, less chest drainage, and higher preoperative hemoglobin and platelet counts. ICU stay, hospital stay, and major complications were comparable between groups, with only one re-opening and two residual shunts in SMS.

**Conclusions:**

RVIAT is a safe, minimally invasive alternative to sternotomy, offering reduced postoperative morbidity and superior cosmetic outcomes. This is particularly advantageous for pediatric and young adult patients.

## INTRODUCTION

Atrial Septal Defect (ASD) is a prevalent congenital heart condition, occurring in 1 to 2 per 1,000 live births and accounting for 25-30% of new diagnoses.^1^,^2^ The first successful closure of an ASD was performed by Lewis and Taufic in 1953, marking a major milestone in congenital heart surgery.^3^ Percutaneous device closure is considered the treatment of choice for children with secundum ASDs, though it has limitations related to defect size, inadequate rims, and complex anatomy.^4^,^5^ Median sternotomy with cardiopulmonary bypass (CPB) remains the standard approach, with reported mortality and morbidity rates of 0%;^5^ however, its scar may be cosmetically unappealing, potentially causing psychological issues, particularly in prepubescent girls.

To address these concerns, many surgical centers now employ minimally invasive (MI) thoracotomy for ASD closure. MI techniques offer benefits such as improved sternal stability, reduced infections, enhanced respiratory function, minimized bleeding, decreased drainage and pain, faster recovery, avoidance of postoperative pectus carinatum, shorter hospital stays, lower costs, and superior cosmetic outcomes.^4^-^6^ Several MI approaches have been described, including partial sternotomy, trans-xiphoid, anterolateral and posterolateral thoracotomy, the right axillary approach, and video-assisted thoracoscopic surgery^4^ advances in cannula design and perfusion technology have further improved safety, reproducibility, and accessibility.^7^

Right thoracotomy and partial sternotomy are now established approaches,^4^ though partial sternotomy leaves a central scar, anterolateral thoracotomy may cause breast and pectoral maldevelopment.^8^,^9^ and posterior thoracotomy offers limited exposure and risks scoliosis.^8^ Schreiber et al. introduced minimally invasive ASD closure via a mid-axillary approach to overcome these limitations.^10^ This area has minimal chest wall muscle coverage, avoids developing breast tissue, and offers a clear view of the atrial septum. We have adopted the right transverse axillary incision for congenital cardiac repairs, providing a discreet, high-axilla scar without anterior chest or back incisions.^11^

RVIAT is an adaptation of the Denis Browne technique introduced in 1952 to prevent long thoracic nerve injury during thoracotomy for PDA repair, preserving the latissimus dorsi and carefully dissecting the long thoracic nerve from the serratus anterior.^12^,^13^ We also prefer a small vertical mid-axillary cut to avoid lymph nodes and cross natural tension lines at a right angle.^5^ The purpose of our study is to compare SMS with RVIAT outcomes for ASD and partial AVSD repair for the first time in a newly established cardiac center in Pakistan, and to evaluate early postoperative outcomes-including pain, recovery time, complications, cosmetic results, hospital stay, and analgesic requirements-between MI thoracotomy and conventional MS.

## METHODOLOGY

This retrospective study included patients who underwent ASD or partial AVSD repair at the Peshawar Institute of Cardiology between June 2024 to July 2025. Participants had documented surgical procedures in their medical records and available follow-up data. A sample of 72 patients with atrial septal defects (ASD) who had undergone surgery at the Peshawar Institute of Cardiology (PIC) were included in this study. Patients were grouped according to the type of surgical procedure they underwent. Each group included 36 patients, with Group-I patients undergoing Standard Median Sternotomy (SMS) and Group-II patients undergoing minimally invasive Right Vertical Infra-Axillary Thoracotomy (RVIAT). Patients diagnosed with ASD were counselled and briefed about the differences between the two surgical methods.

### Ethical Approval:

Peshawar Institute of Cardiology (PIC) provided ethical approval for this study through its Institutional Review Board (IRB), and the research was conducted according to the ethical guidelines of the Helsinki Declaration. All patients, parents or legal guardians of patients provided informed consent before participation. PIC IRB Reference Number: IRC/25/162, Date: 26th February, 2025.

Patients with a prior history of significant cancers, major cardiac surgeries such as coronary artery bypass grafting or valve replacement, along with other conditions unrelated to ASD, were excluded. Moreover, participants with incomplete or insufficient medical records, severe neurological or cognitive deficits, and a history of extreme allergic reactions to anesthesia were also excluded. This was to ensure that the selected patients’ outcomes would genuinely correlate with the impact of the two surgical techniques employed for ASD repair.

We gathered data on several parameters, including the duration of CPB, aortic cross-clamp (ACC) time, mechanical ventilation duration, volume of chest tube drainage, amount of blood transfused, length of hospital stay, length of stay in ICU, preoperative and postoperative hemoglobin and platelet levels, as well as any residual shunt (Table-I).

**Table-I:** Demographic data and diagnosis.

Variables	SMS	(n=36)	RVIAT	(n=36)	p*
	Mean or count	± SD	Mean or count	± SD	
Age (y)	18.39	± 11.65	6.80	± 5.91	<0.001
Gender(m/f)	19/13		22/10		0.475
Weight(kg)	39.42	± 20.33	18.82	± 8.92	<0.001
***ASD types***					
1 Large secondum	28		30		0.58
2 Sinus venosus	6		2		0.3
3 Small secondum	2		4		0.41

For both groups, the preoperative setup involved a thorough clinical evaluation, including detailed history-taking, physical examination, and necessary investigations such as echocardiography, chest X-ray, and blood tests. Patients were typically admitted a day before surgery for preoperative optimization, and fasting guidelines were strictly followed. Prophylactic antibiotics were administered to reduce the risk of surgical site infection.

Anesthesia was induced using standard intravenous agents, followed by endotracheal intubation to secure the airway. Maintenance of anesthesia was achieved through inhalational agents, intravenous anesthetics, and muscle relaxants to ensure optimal surgical conditions. Continuous monitoring of vital signs, including ECG, blood pressure, oxygen saturation, end-tidal CO2, and central venous pressure, was maintained throughout the procedure. Once anesthesia was established, the patient was positioned supine, with the chest prepped and draped in a sterile manner.

For patients with the Right Vertical Infra-Axillary Thoracotomy approach, an incision of 3 cm was made below the right axilla. The latissimus dorsi muscle was retracted backwards, and access to the chest was through the 4th intercostal space. The intercostal muscles were also cut as anteriorly and posteriorly as possible. Two retractors for the chest were placed at right angles to one another in the chest to retract the ribs and soft tissues away from the surgical site. It was followed by aorto-bicaval cannulation. The superior vena cava (SVC) was cannulated with snares put on it, while the inferior vena cava (IVC) was cannulated with snares in place. CPB was established, and cardioplegia was also administered. The right atrium was then opened, and the ASD was closed with an autologous pericardial patch. The patch was sutured with 5-0 prolene in a continuous single layer and reinforced with three additional sutures.

For Median Sternotomy, thymectomy and pericardiotomy were performed. Heparin was administered, and aorto-bicaval cannulation was completed with the superior and inferior vena cava snares held in place. CPB was started with cardioplegia also administered. The right atrium was opened, and the ASD was repaired with an autologous pericardial patch. A continuous single-layer technique was used with 5-0 prolene sutures to attach the patch.

Data were processed with SPSS Ver.27 for Windows for statistical analysis. Quantitative data were expressed as mean ± standard deviation and qualitative data as percentage. An independent sample t-test was used to compare two groups with standard variables, while the chi-squared test was used to compare categorical variables. A statistical significance of <0.05 p-value was presumed. Regression analysis was performed in order to account for age as a possible confounder.

## RESULTS

Out of the 72 patients enrolled in the study, 41 (56.94%) males and 31 (43.05%) females had a mean age of 12.60 ± 10.89 years. There were 19 males and 17 females with a mean age of 18.39 ± 11.65 in Group-I and 22 males and 14 females with a mean age of 6.81 ± 5.99 in Group-II. According to the diagnosis, there were 28 large septum secundum defects, two small septum secundum, and six sinus venosus atrial septal defects in Group-I, and 30 large septum secundum, four small septum secundum, and two sinus venosus defects in Group-II. The demographic data are summarized in Table-I.

In the preoperative period, the differences between the hemoglobin (p = 0.000) and platelet counts (p = 0.001) showed that preoperative hemoglobin was significantly higher in the RVIAT group. The CPB time (p = 0.057) was not significantly different between the two groups. The ACC time (p = 0.021), however, was significantly longer in the SMS group. The severity of heart-failure symptoms according to the NYHA classification also showed no difference (p = 0.629) between the two groups.

In the postoperative period, the ventilation time (p=0.001), amount of chest drainage (p = 0.000), and volume of blood transfusion (p = 0.007) were all significantly higher in the SMS group compared with the RVIAT group. Similarly, the postoperative hemoglobin (p = 0.001) and platelet counts (p = 0.001) showed significant differences, with both parameters being lower in the SMS group. The duration of hospital stay (p = 0.220) and ICU stay (p = 0.913) were not significantly different between the two groups. Upon follow-up, no major complications were observed in either group, except for one case of re-opening in the SMS group, while none of the patients in the RVIAT group required re-exploration (p = 0.314). There was a small residual shunt in two patients from the SMS group (p = 1.000) and none from the RVIAT group. The results are summarized in Table-II.

**Table-II:** Operative and postoperative data.

Variables	SMS (n=36) Group-I mean ± sd	Rviat (n=36) Group-II mean ± sd	P-value	Adjusted p-value
***Operative data***				
Cardiopulmonary bypass (CPB) time (min)	77.47 ± 45.60	61.58 ± 18.51	0.057	
Aortic cross-clamp (ACC) time (min)	46.28 ± 37.94	30.39 ± 13.43	0.021	0.637
Blood transfusion volume (mL)	331.94 ± 204.00	217.64 ± 138.72	0.007	0.032
Chest drainage (mL)	659.72 ± 225.12	483.33 ± 158.80	0.000	0.012
Ventilation time (min)	273.47 ± 108.18	194.53 ± 92.80	0.001	0.088
***Hematologic data***				
Preoperative hemoglobin (g/dL)	13.69 ± 2.08	11.39 ± 2.27	0.000	0.042
Preoperative platelets (×10^9^/L)	244.47 ± 80.92	305.56 ± 74.84	0.001	0.036
Postoperative hemoglobin (g/dL)	10.43 ± 1.72	10.22 ± 1.25	0.564	
Postoperative platelets (×10^9^/L)	150.00 ± 58.57	195.39 ± 51.17	0.001	0.079
***Postoperative outcomes***				
Hospital stay (days)	4.08 ± 1.34	3.75 ± 0.91	0.220	
ICU stay (days)	2.19 ± 1.14	2.17 ± 1.00	0.913	
Residual shunt, n (%)	2 (5.6%)	0 (0%)	1.000	
Re-opening, n (%)	1 (2.8%)	0 (0%)	0.314	

After adjusting for age using multivariable linear regression, some differences between RVIAT and SMS lost statistical significance. Blood transfusion volume, chest drainage, preoperative hemoglobin and platelet counts remained significantly lower in the RVIAT group independent of age. Differences in ventilation time, cross-clamp duration, and postoperative platelet counts were no longer significant after controlling for age, indicating that the originally observed differences were largely age-dependent rather than technique-dependent.

## DISCUSSION

Both techniques were found to be safe and effective, with no significant difference in hospital or intensive care unit stay. However, RVIAT demonstrated advantages in terms of less postoperative chest-tube drainage and reduced blood transfusion requirements. These findings suggest that RVIAT provides equivalent surgical efficacy to SMS while offering a less invasive alternative with favorable short-term postoperative outcomes.

Our results are consistent with those of Yaliniz et al., who reported reduced blood loss, less transfusion requirements, shorter mechanical ventilation, and superior cosmetic satisfaction with RVIAT compared to SMS.^13^ Similar results have also been reported by Yang et al. and Lou et al., supporting minimally invasive approaches as safe and cosmetically superior alternatives to conventional sternotomy for ASD repair.^14^,^15^ In our study, CPB duration did not differ significantly between the two groups, indicating that the minimally invasive approach does not compromise operative safety or efficiency. Although ACC time was longer in the SMS group, this difference lost statistical significance after adjustment for age.

The reduced postoperative blood loss and transfusion requirements observed in the RVIAT group are likely attributable to minimal mediastinal dissection and the absence of sternal marrow bleeding, both inherent to median sternotomy.^9^ The smaller incision and reduced surgical trauma associated with RVIAT may also facilitate faster postoperative recovery, contributing to shorter ventilation duration and improved hematologic recovery. Despite these advantages, comparable ICU and hospital stay between the two techniques indicate both techniques achieve similar postoperative stability.

A notable finding in this study was the significant age difference between the two groups, with the RVIAT patients being considerably younger (6.8 ± 5.9 years) than SMS patients (18.4 ± 11.6 years). Age is a recognized determinant of perioperative outcomes, influencing factors such as blood loss, ventilation time, and recovery duration. Therefore, some of the observed benefits may be partially age-related rather than solely technique-dependent. However, multivariable regression analysis demonstrated that reduced transfusion volume and chest drainage remained independently associated with RVIAT.

The predominance of younger patients in the RVIAT group reflects both anatomical and institutional factors. Pediatric patients offer favorable exposure through a limited infra-axillary incision due to a more compliant chest wall and smaller body size. In addition, cosmetic concerns and parental preference often favor minimally invasive approaches in children and adolescents, particularly females. Similar selection patterns have been reported in early institutional experiences, with minimally invasive congenital cardiac surgery.^13^

An important advantage of RVIAT is its superior cosmetic outcome. Unlike SMS, which leaves a more prominent central scar, the RVIAT employs a minimal right vertical infra-axillary incision that leaves a much less noticeable scar. This is particularly important for pediatric and young adult patients, in whom visible scars may negatively impact body image and self-esteem. Improved cosmetic satisfaction following RVIAT has been associated with better psychological adjustment and overall quality of life, especially among adolescent and young female patients.^8^,^13^,^16^

In real-world situations, the type of surgery for ASD repairs should depend on the patient’s age and gender or the body habitus and anatomy alongside the patient’s personal preference. While SMS remains the gold standard for ASD repairs due to its more straightforward nature, allowing for excellent exposure, particularly in complex cases,^17^ RVIAT provides a valuable alternative when cosmetic outcomes are a priority, especially in pediatric and young female patients.^8^

Our findings add to the growing body of evidence supporting RVIAT as a viable and aesthetically favorable alternative to sternotomy for ASD closure. The concealed axillary incision offers a substantial cosmetic advantage, reducing the psychological and social burden associated with a visible midline scar-an especially relevant factor in young females and pediatric patients in our sociocultural context. Previous studies have also noted improved patient satisfaction and self-image following RVIAT, with equivalent safety and durability compared to SMS.^18^-^20^

**Fig.1:**
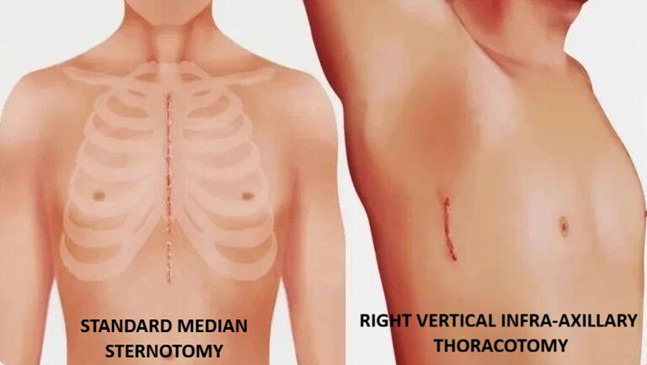
Illustration of SMS and RVIAT.

**Fig.2:**
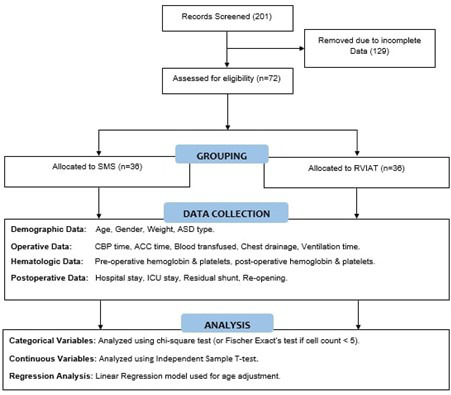
Methodology Summary.

### Limitations:

It was retrospective in design and conducted at a single center, which may limit the generalizability of the findings. The groups were not age-matched, which may have influenced perioperative outcomes in favor of the RVIAT group. Additionally, the follow-up duration was limited to the early postoperative period, and long-term outcomes such as arrhythmia, scar keloid formation, or patch integrity were not evaluated.

## CONCLUSION

RVIAT is a safe, effective, and cosmetically superior alternative to standard median sternotomy for ASD repair. While both approaches yield comparable clinical outcomes, RVIAT offers clear advantages in terms of reduced postoperative morbidity and improved aesthetic satisfaction. With proper patient selection and surgical expertise, RVIAT can be considered the preferred approach for pediatric and young adult patients undergoing ASD repair in resource-limited settings.

### Recommendations:

Future studies with randomized designs and age-matched groups are recommended to confirm the independent effect of the surgical technique on clinical outcomes.
